# Palladium-Gold Modified Ultramicro Interdigital Array Electrode Chip for Nitrate Detection in Neutral Water

**DOI:** 10.3390/mi10040223

**Published:** 2019-03-29

**Authors:** Shanshan Zhao, Jianhua Tong, Yang Li, Jizhou Sun, Chao Bian, Shanhong Xia

**Affiliations:** 1 State Key Laboratory of Transducer Technology, Institute of Electronics, Chinese Academy of Sciences, Beijing 100190, China; ustbzss66@sina.cn (S.Z.); jhtong@mail.ie.ac.cn (J.T.); yangli@mail.ie.ac.cn (Y.L.); sunjizhou628@163.com (J.S.); shxia@mail.ie.ac.cn (S.X.); 2 University of Chinese Academy of Sciences, Beijing 100049, China

**Keywords:** nitrate, ultramicro electrode, Pd-AuNPs, neutral water

## Abstract

An ultramicro interdigital array electrode modified by palladium-gold was developed for nitrate detection in neutral water. The ultramicro interdigital array electrode was fabricated based on silicon substrate by Micro Electro-Mechanical System (MEMS) technique. The nanostructured palladium-gold (Pd-AuNPs) composite sensing film was electrodeposited on the surface of a working electrode by electrochemical method. The synergistic effect of Pd-AuNPs composite was investigated and its enhancement of the catalytic activity and stability was revealed. The Pd-AuNPs modified electrode showed good linearity (R^2^ = 0.99) from 1 mg/L to 15 mg/L (as N) for nitrate determination in a neutral water environment (pH = 7.2), with a sensitivity of 4.7 μA·mg^−1^·L. The results showed that the developed Pd-AuNPs-modified ultramicro interdigital array electrode chip can achieve sensitive and environmentally-friendly detection for nitrate in neutral water.

## 1. Introduction

The quality of water seriously affects the environment and people’s health. With the development of industry and agriculture, the pollution of water has been more and more serious. Nitrate is one of the common pollutants in water. The accumulation of nitrate in water can lead to eutrophication of water bodies. The reduction products of nitrite may also cause serious human diseases [1]. Therefore, in order to prevent the excessive nitrate in the water from harming the environment and human health, the detection for nitrate in water is of great necessity and significance. The world health organization has recommended that the nitrate level in potable water should not exceed 11 mg·L^−1^ (as N) [2].

There are many kinds of method to detect nitrate that have been revealed and experimented, such as spectroscopy, chromatography, capillary electrophoresis, and electrochemical methods [3]. Although all of the methods above can achieve the detection of nitrate under different requirements, the former three have many limits in quick detection because of the bulky and expensive devices and the sophisticated operation. The electrochemical method has advantages in water quality monitoring because its devices are easy to operate, and easy to integrate and automate. Therefore, the electrochemical method can be an effective way to achieve nitrate concentration detection. 

Nitrate electrochemical sensors based on carbon nanotubes and copper nanomaterials were fabricated by Wenjing Lu [4]. The experimental process showed that the pH value of the backup electrolyte needs to be adjusted to the acidic range of about 2.0 with concentrated sulfuric acid. Pb-Sn composite sensing film modified electrode chips were developed by Yexiang Fu [5]. Thermal annealing of Cu nanowires was used to detect nitrate in the acidic environment by Jihong Liang et al. [6]. Though high sensitivity and good stability were achieved, it was also necessary to adjust the pH value to the acidic environment before the detection.

What has been discussed above indicated that the current detection of nitrate concentration usually needs to be carried out in an acidic solution environment. The added acid medium causes secondary pollution to the environment, and the pretreatment process of the test is complicated. A microfluidic impedance nitrate sensor using a graphene oxide (GO) nano-sheet and poly(3,4-ethylenedioxythiophene) nanofiber (PEDOT-NFs)-enabled electrochemical sensing interface was developed to be used in soil environments by Ali et al. [7]. Silver and carbon ultramicro electrodes (UMEs) modified by the silver nanoparticles were developed by Zhad et al. [8] without adjusting the pH value. However, the stability and sensitivity of this silver-modified ultramicro electrode array should be improved. Therefore, a kind of ultramicro interdigital array electrode modified with a Pd-AuNPs composite sensitive membrane was developed in this paper for the determination of nitrate in a neutral water environment. There are studies about nanoparticles, composite metal film, and micro electrodes [9,10,11,12,13,14,15,16,17,18,19,20,21,22,23,24,25,26], in which we can learn that the current response and sensitivity to nitrate in a neutral environment were improved by ultramicro electrode array and the synergistic effect of palladium and Au composite metal materials. 

## 2. Materials and Methods 

### 2.1. Reagents 

All solid reagents were of analytical grade and were used without further purification. Ethanol, HAuCl_4_, and sulfate acid were obtained from Beijing Chemical Works (Beijing, China). NaCl and KNO_3_ were obtained from Sinopharm Chemical Reagent Co. Ltd. (Shanghai, China). PdCl_2_ was obtained from Sigma-Aldrich (Saint Louis, MO, USA). Water used in the experiment was all deionized water with a resistivity of 18 MΩ·cm obtained from Millipore Direct-Q 3 UV system (Merck Millipore Co., Billerica, MA, USA).

### 2.2. Apparatus

The electrochemical experiments were performed with a Gamry Reference 600 electrochemical analyzer. The KCl saturated Ag/AgCl electrode acted as a reference electrode. A working electrode and counter electrode were integrated on the ultramicro interdigital array electrode chip. Scanning electron microscopy (SEM) images of the deposited layer were performed by using an S-4800 field emission scanning electron microscope (FESEM) produced by Hitachi (Tokyo, Japan).

### 2.3. Methods

Firstly, the ultramicro interdigital array electrodes were fabricated by Micro Electro-Mechanical System (MEMS) technique. Then, the ultramicro interdigital array electrodes were cleaned by ultrasonic method and scanned in dilute sulfuric acid by cyclic voltammetry method with a voltage of −0.2 V–1.5 V for 5 circles. After that, the palladium-nanostructured gold (Pd-AuNPs) composite sensing film was electrodeposited on the working electrode by constant voltage method. The metal palladium was modified with a voltage of −0.4 V for 100 s, then the gold nanoparticle was modified with a voltage of −0.2 V for 300 s. All the SEM images were obtained by FESEM. The concentration of potassium nitrate ion was detected by the Pd-AuNPs-modified ultramicro interdigital array electrode chip by linear sweep voltammetry with a voltage of 0 V–−0.7 V. 

## 3. Experimental and Results

### 3.1. Fabrication of Nitrate Sensing Electrode Chip

The electrochemical experiments in this paper were all performed using conventional 3 electrodes electrochemical system consisting of working electrode (WE), counter electrode (CE), and reference electrode (RE). In this paper, the ultramicro electrode array prepared by Micro Electro-Mechanical System (MEMS) technique was used as the electrochemical electrode to take advantage of its electrochemical analysis, miniaturization, and integration. One dimension of the ultramicro electrode is smaller than the thickness of the diffusion layer, which is usually within the range of micron or nanometer. It has the advantages of high mass transfer rate and high signal-to-noise ratio. Combining a single ultramicro electrode in the form of an array not only retains the advantages of the ultramicro electrode, but also improves the current response.

**Figure 1:**
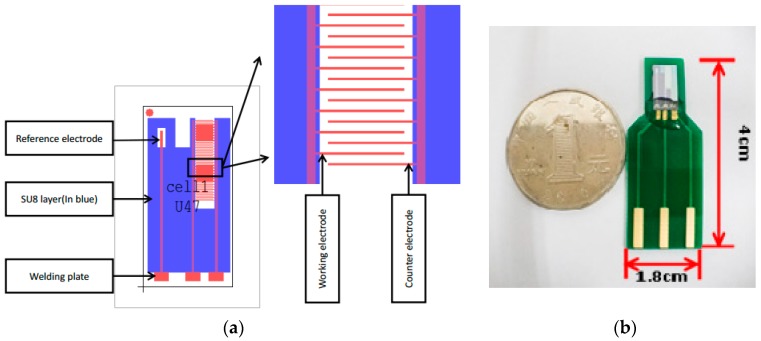
(**a**) The structure diagram and (**b**) the picture of the ultramicro interdigital electrode array chip.

The structure diagram of the ultramicro interdigital array electrode is shown in Figure 1a. There are 30 pairs of working electrodes and counter electrodes on the same chip. The length of the working electrode is 1 mm, the width is 15 μm, and the effective working electrode area is 0.45 mm^2^. The picture of one ultramicro interdigital electrode array chip is shown in Figure 1b. The fabrication process involves oxidation on the silicon substrate, followed by lithography, sputtering, and stripping to obtain the pattern of ultramicro interdigital electrode arrays. 

### 3.2. Preparation of Pd-Aunps Composite Sensing Film

**Figure 2:**
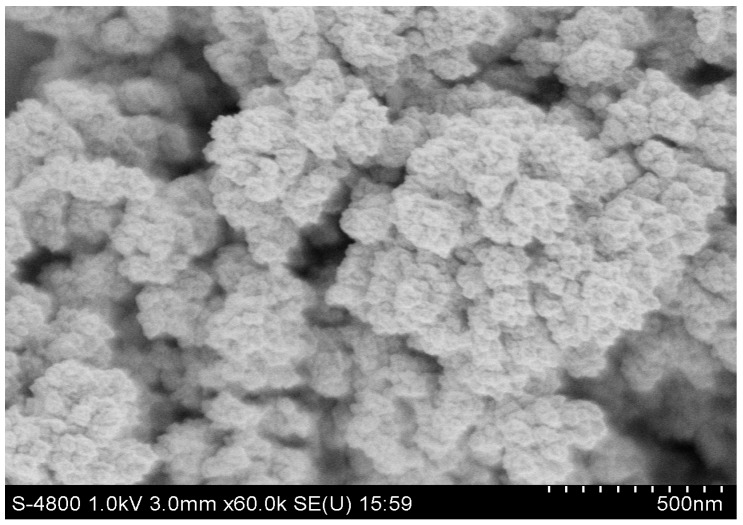
The SEM image (× 60K) of the metal Pd.

The obtained ultramicro interdigital electrode chip was cleaned by cyclic voltammetry (CV) method scanning in 0.05 M dilute sulfuric acid. The scanning voltage range was from −0.2 V to 1.5 V and the scan rate was 50 mV/s. Metal palladium was electrodeposited on the platinum-based ultramicro interdigital electrode array by using the potentiostatic method. Firstly, palladium was electrodeposited on the working electrode at a fixed potential of −0.4 V for 100 s. The concentration of the electrolyte for palladium is 20 mmol/L, which is obtained by dissolving PdCl_2_ in NaCl of 1 mol/L. The SEM image (× 60K) of the metal palladium is shown in Figure 2, in which the spherical metal palladium can be seen clearly. 

**Figure 3:**
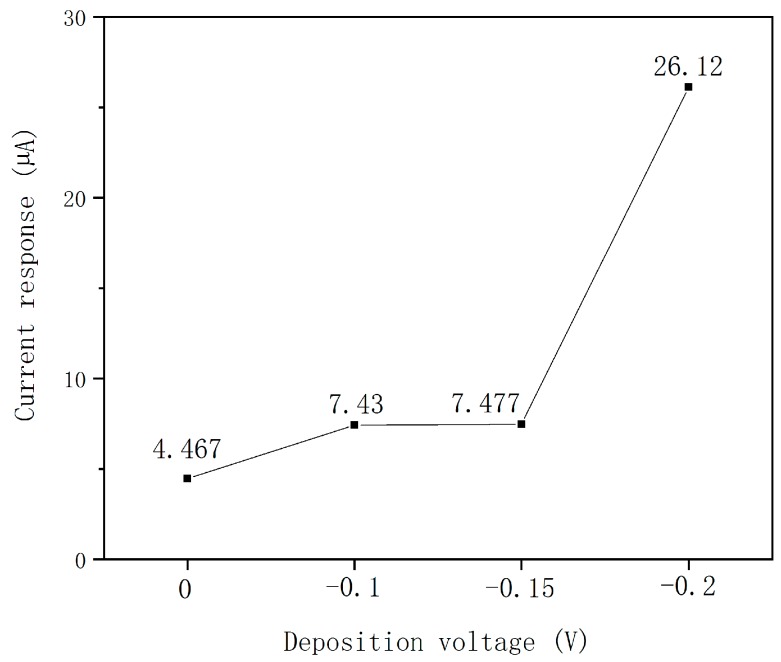
Current response of the Pd-AuNPs modified with different deposition voltages.

After the palladium was modified on the electrode, the AuNPs was electrodeposited on the palladium layer with a fixed potential of −0.2 V for 300 s. The electrolyte for AuNPs is 10 mmol/L of HAuCl_4_. The deposition voltage was optimized by electrochemical linear sweep voltammetry (LSV). Firstly, five kinds of deposition voltage were chosen according to the electrochemical property of Au in 10 mmol/L of HAuCl_4_. The voltages were 0 V, −0.1 V, −0.15 V, −0.2 V, and −0.3 V. Then, the five chips modified with Pd-AuNPs under different deposition voltages were used to detect the neutral potassium nitrate solution with a concentration of 1 mg/L. Finally, the current responses obtained above were compared together (as seen in Figure 3). The results showed that the current response is highest when the deposition was −0.2 V. The AuNPs aggregated when the deposition was −0.3 V and the WE and CE were connected together, under which condition detection cannot go on. Therefore, −0.2 V was chosen to be the deposition voltage of AuNPs.

**Figure 4:**
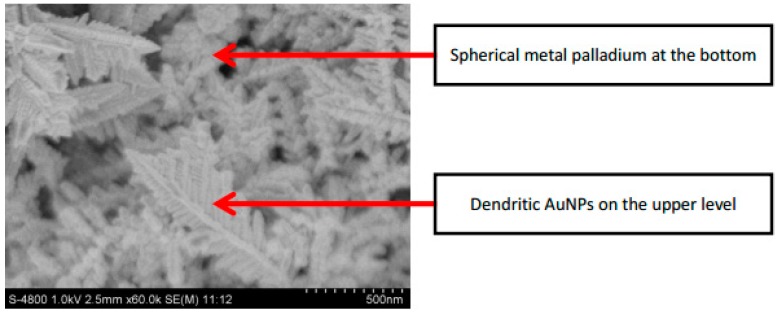
SEM images of Pd-AuNPs composite sensing film with magnification of 60K.

The SEM image (× 60K) of the Pd-AuNPs composite sensitive film is shown in Figure 4, in which the spherical metal palladium at the bottom and the dendritic AuNPs on the upper level can be seen. The average diameter of metal Pd is about 1.4 μm and the average branch width of metal AuNPs is about 90 nm.

### 3.3. Detection Performance of the Ultramicro Interdigital Electrode Chips Modified by Pd-Aunps Composite Sensitive Membrane for Nitrate

#### 3.3.1. Characterization of Pd-Aunps to Nitrate

**Figure 5:**
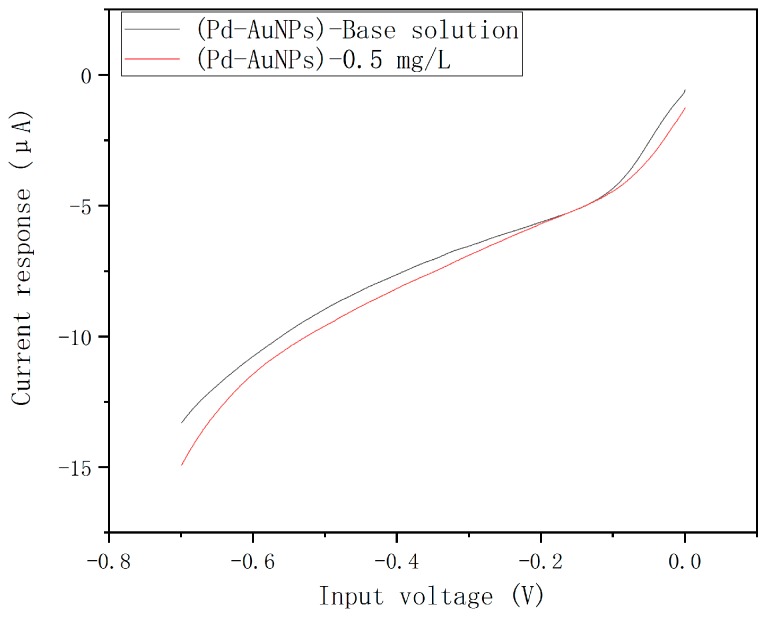
The linear scanning curves of the Pd-AuNPs composite sensing film modified chip in the base solution and the nitrate solution with a concentration of 0.5 mg·L^−1^.

Three chips were chosen to do the experiment below. Firstly, all the chips were cleaned through the same cleaning process. Then, the working electrodes of these chips were deposited with palladium, Au, and Pd-AuNPs composite sensing film according to the optimal parameters above, respectively. Next, all the chips were stabilized by linear sweep voltammetry multiple times in the base solution of phosphate buffer, which was composed of disodium phosphate solution and sodium dihydrogen phosphate solution. The pH value of the phosphate buffer was 7.2. Linear sweep voltammetry was chosen to detect the nitrate in water because it is a commonly used method for electrochemical detection of nitrate [5,6,27,28]. The detection was performed in nitrate solution with a concentration of 0.5 mg·L^−1^. The linear scanning curves of the Pd-AuNPs composite sensing film modified chip in the base solution and the nitrate solution are shown in Figure 5. Due to the different current responses of these three chips in the base solution, for comparison the current response for nitrate solution was recorded as the current difference between the nitrate solution and the base solution. We used five measurements to calculate each standard deviation. The time used to take the experiments from the first measurements to the last was about 120 s. The results were recorded in Table 1. The results indicated that the chip modified by Pd-AuNPs composite sensing film showed the highest sensitivity and good repeatability with nitrate.

The composite nanoparticle has the advantages of large surface area, surface effect, and small size effect. The excellent performance of the composite material is not only because of the increase in specific surface area, but also the hydrogen evolution of palladium, which is helpful in stabilizing the sensing activity of Au for nitrate reduction. The role of gold in the bimetallic is to reduce the nitrate to nitrite by direct redox reaction, while the metal palladium maintains the catalytic metal gold in its metallic state by adsorbing hydrogen. Therefore, the close contact of the two metals plays an important role in increasing the activity of the catalyst to degrade nitrate [29,30]. The synergistic effect of palladium and Au nanoparticles can effectively improve the catalytic performance of nitrate towards the sensing film. Finally, the detection performance can be effectively improved by combining the excellent properties of the ultramicro electrode array and the composite nanoparticle. 

#### 3.3.2. Nitrate Determination with the Pd-Aunps Modified Chip

**Figure 6:**
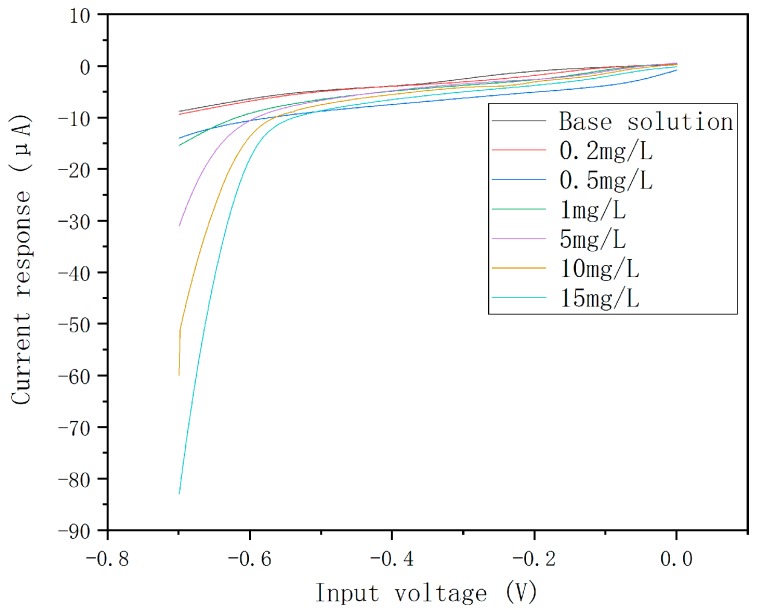
The linear sweep voltammetry plots for the nitrate of the chips modified by Pd-AuNPs composite sensing film.

Linear sweep voltammetry was used for the current response characteristic test of the Pd-AuNPs to nitrate. The linear sweep voltammetry plots are shown in Figure 6.

**Figure 7:**
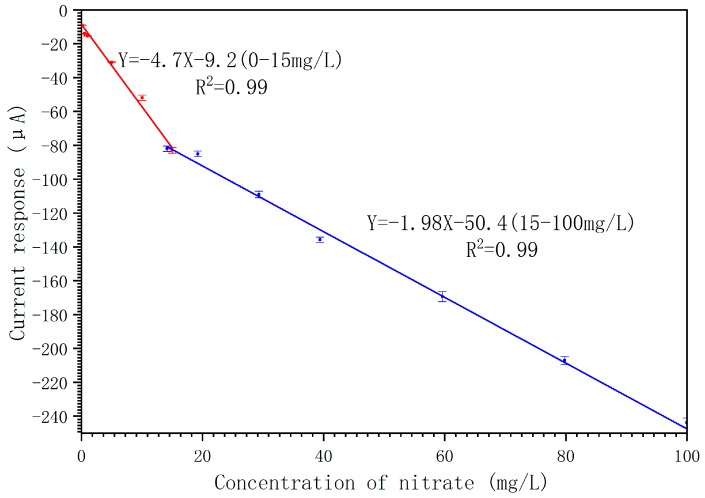
The response curve of the chips modified by Pd-AuNPs composite sensing film.

The response curve is shown in Figure 7. The results indicate that the ultramicro interdigital electrode chip modified by Pd-AuNPs composite sensing film can be used for the detection of nitrate ion concentration in neutral water with a pH of 7.2 and the linearity is good, at 0.99. The sensitivity is 4.7 μA·mg^−1^·L when the concentration of the nitrate is lower than 15 mg·L^−1^, and the detection limit is 0.074 mg·L^−1^ (3δ, δ is the ratio of signal-to-noise). The sensitivity is 1.98 μA·mg^−1^·L in the range of 15–100 mg·L^−1^. The results showed that the chips modified by Pd-AuNPs composite sensing film can also be used for the determination of nitrate with high concentration, though the sensitivity is relatively low. 

#### 3.3.3. Anti-Interference Test

**Figure 8:**
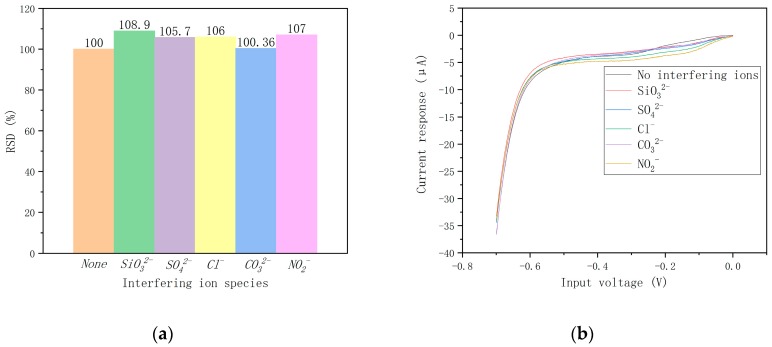
(**a**) Relative standard deviations caused by different interference ions. (**b**) The response curves for nitrate detection with different interfering ions.

Firstly, 10 mg·L^−1^ potassium nitrate solution (with PB as buffer) and 100 mg·L^−1^ interference solution (with water as buffer) were prepared. The interference ions are silicate-containing ion SiO_3_^2−^, sulfate ion SO_4_^2−^, chloride ion Cl^−^, carbonate ion CO_3_^2−^, and nitrite ion NO_2_^−^. Then, we mixed the potassium nitrate solution, interference solution, and deionized water to obtain the potassium nitrate solutions containing various interfering ions. Chips modified by Pd-AuNPs composite sensing film were used to detect the potassium nitrate solution with a non-interfering ion and five kinds of interference ions, respectively. The influence of each interference ion on the current response of nitrate ions was detected. The relative standard deviations caused by the presence of interfering ions and the response curves for nitrate detection with different interfering ions are shown in Figure 8a,b. The results indicate that ultramicro electrode chips modified by the composite sensing film in this paper have certain anti-interference ability, which can ensure that the deviations caused by common interference ions were all less than 10%.

#### 3.3.4. Actual Water Sample Test

Three actual water samples were taken for detection, all of which were tap water. The water samples were sent to the detection institution to detect the concentration of nitrate. Then, we used an ultramicro interdigital electrode chip modified by Pd-AuNPs composite sensing film to detect the same samples. The results were compared with relative deviation, as shown in the Table 2. The results have shown that the relative deviation is all lower than 20%, which means the detection performance of the ultramicro interdigital electrode chip modified by Pd-AuNPs composite sensing film in actual samples can satisfy the demands.

## 4. Conclusion and Discussion

The ultramicro interdigital electrode chip modified by Pd-AuNPs composite sensing film was prepared for the detection of nitrate ion concentration in neutral water. The ultramicro array electrode was combined with composite metal sensing film. The synergistic effects of the palladium and Au nanoparticle increased the surface area of the sensing film and the catalytic activity was enhanced. The sensitivity and stability of the detection for nitrate in neutral water were improved. This paper provided a feasible method for the sensitive detection of nitrate in a neutral water environment.

In the future, the detection performance and the ability to detect actual water samples of the ultramicro interdigital electrode chip modified by Pd-AuNPs composite sensing film needs to be further optimized. 

## Figures and Tables

**Table 1:** The responses to nitrate of the chips modified with different sensing film.

Sensing Film	The Current Response (μA)	Relative Standard Deviation (%)
Palladium	unstable	–
Au nanoparticle	0.490	6.00
Pd-AuNPs	1.625	4.50

**Table 2:** The results of the actual water sample test.

Sample	Detection Results of Institution (mg/L)	Detection Results of this Paper (mg/L)	Relative Deviation (%)
**Sample1**	1.17	1.40	19.7
**Sample2**	1.19	1.41	18.5
**Sample3**	1.20	1.36	13.3

## References

[1] http://www.mee.gov.cn/hjzl/zghjzkgb/lnzghjzkgb/201606/P020160602333160471955.pdf.

[2] (1993). Guidelines for Drinking Water Quality Recommendations.

[3] Li W. (2013). Evaluation of measurement uncertainty of water quality—Determination of total nitrogen—Alkaline potassium persulfate digestion UV spectrophotometric method. Northern Environment.

[4] Pan D., Lu W., Wu S., Zhang H., Qin W. (2012). In situ spontaneous redox synthesis of carbon nanotubes/copper oxide nanocomposites and their preliminary application in electrocatalytic reduction of nitrate. Mater. Lett..

[5] Fu Y., Bian C., Kuang J., Li Y., Xia S. (2016). Nitrate sensing based on Pd-Sn bimetallic composite: A comparison between a bulk electrode and a microband electrode array. IET Micro Nano Lett..

[6] Liang J., Zheng Y., Liu Z. (2016). Nanowire-based Cu electrode as electrochemical sensor for detection of nitrate in water. Sens. Actuat. B Chem..

[7] Ali M.A., Jiang H., Mahal N.K., Weber R.J., Kumar R., Castellano M.J., Dong L. (2017). Microfluidic impedimetric sensor for soil nitrate detection using graphene oxide and conductive nanofibers enabled sensing interface. Sens. Actuat. B Chem..

[8] Zhad H.R.L.Z., Lai R.Y. (2015). Comparison of nanostructured silver-modified silver and carbon ultramicroelectrodes for electrochemical detection of nitrate. Analyt. Chim. Acta.

[9] Tatka L., Kim U. Affordable, rapid, electrochemical nitrate detection towards point-of-use water quality monitoring. Proceedings of the 2016 IEEE Global Humanitarian Technology Conference (GHTC).

[10] Alahi M.E.E., Xie L., Mukhopadhyay S., Burkitt L. (2017). A Temperature Compensated Smart Nitrate-Sensor for Agricultural Industry. IEEE Trans. Industr. Electron..

[11] Paixão T.R.L.C., Cardoso J.L., Bertotti M. (2007). Determination of nitrate in mineral water and sausage samples by using a renewable in situ copper modified electrode. Talanta.

[12] Gamboa J.C.M., Peña R.C., Paixão T.R.L.C., Bertotti M. (2009). A renewable copper electrode as an amperometric flow detector for nitrate determination in mineral water and soft drink samples. Talanta.

[13] Pan D., Lu W., Zhang H., Zhang L., Zhuang J. (2013). Voltammetric determination of nitrate in water samples at copper modified bismuth bulk electrode. Int. J. Environm. Analyt. Chem..

[14] Casella I.G., Contursi M. (2014). Highly dispersed rhodium particles on multi-walled carbon nanotubes for the electrochemical reduction of nitrate and nitrite ions in acid medium. Electrochim. Acta.

[15] Bagheri H., Hajian A., Rezaei M., Shirzadmehr A. (2017). Composite of Cu metal nanoparticles-multiwall carbon nanotubes-reduced graphene oxide as a novel and high performance platform of the electrochemical sensor for simultaneous determination of nitrite and nitrate. J. Hazard. Mater..

[16] Szunerits S., Boukherroub R. (2008). Investigation of the electrocatalytic activity of boron-doped diamond electrodes modified with palladium or gold nanoparticles for oxygen reduction reaction in basic medium. C. R. Chim..

[17] Jiang J., Zhang L., Shanbhag V. (2013). Improving Electrochemical Sensitivity of Silver Electrodes for Nitrate Detection in Neutral and Base Media through Surface Nanostructuration. J. Electrochem. Soc..

[18] Da Silva I.S., de Araujo M.R., Paixão T.R.L.C., Angnes L. (2013). Direct nitrate sensing in water using an array of copper-microelectrodes from flat flexible cables. Sens. Actuat. B Chem..

[19] Mahmoudian M.R., Alias Y., Basirun W.J., Woi P.M., Jamali-Sheini F., Sookhakian M., Silakhori M. (2015). A sensitive electrochemical nitrate sensor based on polypyrrole coated palladium nanoclusters. J. Electroanalyt. Chem..

[20] Li Y., Sun J., Bian C., Tong J., Xia S. (2010). Electrodeposition of Copper Nano-clusters at a Platinum Microelectrode for trace nitrate determination. Key Eng. Mater..

[21] Gokhale A.A., Lu J., Weerasiri R.R., Yu J., Lee I. (2015). Amperometric Detection and Quantification of Nitrate Ions Using a Highly Sensitive Nanostructured Membrane Electrocodeposited Biosensor Array. Electroanalysis.

[22] Ali M.A., Jiao Y., Tabassum S., Wang Y., Jiang H., Dong L. Electrochemical detection of nitrate ions in soil water using graphene foam modified by TiO2 nanofibers and enzyme molecules. Proceedings of the 2017 19th International Conference on Solid-State Sensors, Actuators and Microsystems (TRANSDUCERS).

[23] Alahi M.E.E., Xie L., Zia A.I., Mukhopadhyay S., Burkitt L. Practical nitrate sensor based on electrochemical impedance measurement. Proceedings of the 2016 IEEE International Instrumentation and Measurement Technology Conference Proceedings.

[24] Bui M.-P.N., Brockgreitens J., Ahmed S., Abbas A. (2016). Dual detection of nitrate and mercury in water using disposable electrochemical sensors. Biosens. Bioelectron..

[25] Li Y., Du Q., Liu T., Peng X., Wang J., Sun J., Wang Y., Wu S., Wang Z., Xia Y. (2013). Comparative study of methylene blue dye adsorption onto activated carbon, graphene oxide, and carbon nanotubes. Chem. Eng. Res. Des..

[26] Hu J., Sun J., Bian C., Tong J., Xia S. (2013). 3D dendritic nanostructure of silver-array: Preparation, growth mechanism and application in trace nitrate sensor. Electroanalysis.

[27] Davis J., Moorcroft M.J., Wilkins S.J., Compton R.G., Cardosi M.F. (2000). Electrochemical detection of nitrate and nitrite at a copper modified electrode. Analyst.

[28] Stortini A.M., Moretto L.M., Mardegan A., Ongaro M., Ugo P. (2015). Arrays of copper nanowire electrodes: Preparation characterizationand application as nitrate sensor. Sens. Actuat. B Chem..

[29] Deganello F., Liotta L.F., Macaluso A., Venezia A.M., Deganello G. (2000). Catalytic reduction of nitrates and nitrites in water solution on pumice-supported Pd–Cu catalysts. Appl. Catal. B Environment..

[30] Yang D., Feng W., Wu G., Li L., Guan N. (2011). Nitrate hydrogenation on Pd–Cu/TiO_2_ catalyst prepared by photo-deposition. Catal. Today.

